# Childhood Experiences and Sporting Event Visitors’ Preference for Unhealthy versus Healthy Foods: Priming the Route to Obesity?

**DOI:** 10.3390/nu10111670

**Published:** 2018-11-05

**Authors:** Joerg Koenigstorfer

**Affiliations:** Department of Sport & Health Management, Technical University of Munich, Uptown Munich—Campus D, Georg-Brauchle-Ring 60/62, 80992 Munich, Germany; joerg.koenigstorfer@tum.de; Tel.: +49-89-289-24558

**Keywords:** childhood memories, sport spectators, sport games, sporting events, music events, food consumption, out-of-home eating, food healthiness

## Abstract

To date, there is little knowledge about how experiences in childhood frame adults’ food and drink consumption patterns in the context of attending sporting events as spectators. Therefore, the goal of this study was to explore the childhood memories of adults when they visited sporting events and find out whether and why this particular setting makes individuals indulge in unhealthy food. The study comprises two components: Study 1 and Study 2. In Study 1, 30 individuals recalled their childhood experiences of sport stadium visits at the age of ten years or younger. Inductive coding of the stories revealed that on-site enjoyment is an important factor that may lead to unhealthy food consumption. In Study 2 (*n* = 240), the effect of enjoyment on the intentions to eat unhealthy versus healthy food at sporting events was tested empirically and contrasted with two other leisure-time activities. The results of the experiment revealed that it is not enjoyment, but the visit to sporting or music events (versus a flea market) that increased the preference for unhealthy versus healthy foods. Implications to decrease (increase) the preference for unhealthy (healthy) food in these particular settings against the background of childhood experiences can be drawn.

## 1. Introduction

What food and drinks do you associate with your family’s most recent visit to a baseball game? It would be of little surprise if you mentioned that your children had hotdogs and soft drinks. It is noteworthy that these foods are in conflict with the diets of athletes whom you and your family members were following during the course of the game (assuming that athletes stick to the recommendations of sport nutritionists, e.g., [1]).

In consumer behavior and food research, it is well known that childhood experiences influence both purchase behaviors and consumption behaviors in adulthood [2,3]. The attendance of sporting events as a spectator, however, has not been researched extensively yet, particularly with regard to people’s eating and drinking behaviors that are potentially influenced by childhood experiences. This is despite the fact that the food that is provided to children (and adults) when attending sporting events has been subjected to criticism, particularly because of its low nutritional value and high calorie density [4,5]. If childhood memories about the association between food and sporting event attendance as spectators are important, it is crucial that children are exposed to healthy, and not to unhealthy, food environments today (arguing from the perspective of public policy), because these contextual factors influence behavioral patterns later in life.

### 1.1. Food Provision to Sporting Event Spectators

At sporting events, concession stands offer various food and drink options to spectators, and many spectators consume food and drinks before or while following a sporting event. Often, sponsorship and the exclusiveness rights that go along with the sponsorship determine the kind of food and drink provision [6,7], and sport spectators can choose from those options that are made available to them [8].

Within the context of the attendance of sporting events, four factors characterize the market for food and drinks, including the physical facility (i.e., the built environment for food and drink provision/availability as well as for eating and drinking), group experience (i.e., the influence of peer groups, such as spouses, friends, siblings, and strangers, on food and drink choices as well as eating and drinking), history and tradition factors (e.g., having hotdogs at half time for nostalgic reasons), and rituals (e.g., scripts that sporting event visitors follow when attending a game, such as drinking beer when tailgating before the game) [9]. These characteristics affect what and how much people eat and drink.

Marketers make use of the connection between the various food and drink options and the consumers following sporting events in their leisure time, particularly for the promotion of unhealthy food and drinks, targeting children and adolescents [6], generation Y consumers [10], sport gamblers [11], and sport stadium visitors [4], for example. In sport stadiums, concession stands contribute to the perception and appeal of the ‘sportscape’, that is, the stadium as an environment in which services are provided and value is (co-)created [12], and the concession stands may become part of the psychological associations within this environment, a ‘home ground’, a beloved place to sporting event spectators (sometimes called ‘topophilia’ [13,14]).

### 1.2. Childhood Memories about Eating and Drinking When Following Sporting Events On-Site

Attending sporting events is a common leisure time activity for families with children around the world [15,16]. The sensory experience with certain foods and drinks at sporting events can then influence children’s preferences for food and drinks (particularly sweet and salty food and sweet drinks) or their avoidance of food and drinks that they do not like or know of [17,18]. As dietary habits and the acceptance of certain foods and drinks and amounts influence individuals’ health and important health determinants later in life [17], the sporting event setting may be of relevance in this context [5,6,7,8].

The emotions associated with the food and drinks provided and consumed at sporting events relate to the discrete emotions that have been generally studied in food decision-making models (e.g., enjoyment), such as in the goal conflict model of eating [19]. According to the model, there is a conflict between the enjoyment goals of eating and the cognitive representations of weight control (or healthy eating). At sporting events, spectators have been reported to enjoy foods and drinks such as meat pies, burgers, fries, popcorn, and soft drinks [4]—options that are considered to contribute to overweight and obesity because of their low nutritional value, high calorie density, and large portion sizes, which often go along with relatively high consumption volumes (and calorie intake). Yet, some stadiums also have healthy food items on offer, such as salad (e.g., from the stadium’s rooftop garden at Fenway Park, Boston, MA, USA) and cauliflower sandwiches (Wrigley Field, Chicago, IL, USA). The memories about eating and drinking may then influence consumers’ preferences for certain food and drinks at sporting events.

### 1.3. Aims and Research Goals

The present study aimed to capture and categorize the food- and drinks-related memories of adults when they visited sporting events in their childhood. Since enjoyment might be a central emotion (be it related to the game itself, such as when a goal is scored, or the experience in the stands, such as when cheering for the team, being with family and friends, as well as eating and drinking), the present study also addressed the relevance of enjoyment in this context. In particular, it aimed to find out whether the attraction to unhealthy food and the avoidance of healthy food observed in sporting event visitors is merely due to the activation of enjoyment goals (and thus should be replicable across contexts if enjoyment goals are activated) or whether sporting event visits can lead spectators to indulge or to control the effect of enjoying the leisure time activity on the intentions to indulge. Thus, the research questions that guided the research are as follows:

RQ 1 What are the childhood memories of adults when they visited sporting events, particularly in relation to food and drinks?
RQ 2 What is the influence of the particular sporting event setting and the enjoyable experience of the event visit on individuals’ intentions to indulge in unhealthy foods versus healthy foods?

In what follows, two studies are presented: Study 1, which aimed to answer RQ 1, and Study 2, which aimed to answer RQ 2.

## 2. Study 1

### 2.1. Materials and Methods

Thirty informants (15 women) were recruited via Amazon’s Mechanical Turk and took part in the study in exchange for monetary compensation (M [mean] = 34.8 years, SD [standard deviation] = 8.9). Informants were only interviewed when they could recall a professional sporting event that they had visited at the age of 10 years or younger [20,21]. They were US residents and recalled the first time they visited a sporting event, at the age between four and 10 years, according to the written interviews with the informants. The following sports were mentioned: baseball, basketball, football, and ice hockey.

Similar to the procedure in Braun–Latour et al.’s study [3], informants were asked to write down a memory story of their earliest childhood memory. MAXQDA software (VERBI, Berlin, Germany) was used to inductively code the response, based on content analysis procedures [22]. Categories were withdrawn directly from the raw data. The categories that were extracted were treated as content units. The coded content units consisted of three categories (level a) and eleven subcategories (level b). Two coders performed the coding; inter-rater reliability was satisfied with Cohen’s κ = 0.82.

### 2.2. Results and Discussion

In the memory stories, a number of different themes were mentioned. The inductive coding revealed three categories (level a): individual experiences of the visit; external factors related to the visit; social factors related to the visit. Table 1 shows the eleven subcategories (level b) of the three categories and some example statements.

Every informant referred to food and drinks in the memory task, an indicator that there is a close association between food and drinks and sporting event visits in childhood. Within the emotions subcategory, enjoyment and enjoyment-related facets, such as happiness and excitement, were dominant themes in the informants’ memory stories. This supports the assumption that enjoyment is central to the attendance of a sporting event in childhood. The data further revealed that enjoyment relates to many aspects: the game itself, the players, the audience, and the stadium, for example. Most importantly to the present study, informants also made connections between enjoyment and the food experience. For example, informant 11 made the following statement: “The hotdogs there smelled and tasted much better than the ones my mom would make at home. I was not a big hotdog fan, but I did really enjoy the ones there.” Informant 26 highlighted that her food preferences are still the same today: “The hotdog was definitely the classic, made up the whole experience, and I still love pretzels today.” The foods that were mentioned in the stories were the following: burger, chili, french fries, hotdogs, nachos, pizza, and snacks (cotton candy, crackers, peanuts, and popcorn were mentioned explicitly). The drinks that were mentioned were the following: coke, soda, water, and hot chocolate. Table 1 shows some further example statements taken from the memory stories.

To find out whether enjoyment generally predicts intentions to eat unhealthy foods (e.g., the types of foods mentioned in the memory stories) versus healthy foods and whether the sporting event context provides a unique setting to spectators in terms of the preference for unhealthy versus healthy food consumption, Study 2 was conducted. In the study, the visit to a sporting event was contrasted with other leisure time activities, that is, the visit to a music event and the visit to a flea market. This allowed drawing conclusions about the peculiarities of the different settings in which food is consumed. The study exclusively looked at intended food (but not drink) consumption.

## 3. Study 2

### 3.1. Materials and Methods

Two hundred forty students (149 women) were recruited on a university campus and took part in the study in exchange for monetary compensation (M = 26.6 years, SD = 8.5). The experimental study applied a 2 × 2 design, manipulating enjoyment (high versus low) and event type (sporting versus music event) between participants, and a control group was added as a fifth experimental condition (i.e., the visit to a flea market with low enjoyment). In the study, the participants were randomly assigned to one of the five conditions.

The participants first read a description of an event visit (Appendix A) and they were asked to imagine that they would visit the event. After they read the description, they were asked to rate the likeliness to consume 15 different foods on a scale anchored at 1 (“I would not eat this food at all”) and 10 (“I would definitely eat this food”), including both healthy and unhealthy foods ([23,24]; Appendix B). They were pretested to represent healthy and unhealthy foods, which were presented in a random order. An overall score for the preference of unhealthy foods versus healthy foods was computed (with reverse-coded items for healthy options) (α = 0.76).

Beside these intentions, the survey assessed sociodemographics and an item that was used to assess whether the experimental manipulation worked or not (“How enjoyable do you rate the visit to the sporting event (or music event or flea market)?”, anchored at 1 = “Not enjoyable at all” and 7 = “Very enjoyable”). A funneled debriefing was applied at the end of the study, which revealed that none of the participants guessed the research questions of the study.

### 3.2. Results and Discussion

The experimental manipulation worked as intended: when the event visit was described as highly enjoyable, enjoyment was rated higher (M = 5.69, SD = 1.14) compared to when the visit was not described as highly enjoyable (M = 4.24, SD = 1.55; *t* (238) = 8.35, *p* < 0.001).

**Figure 1:**
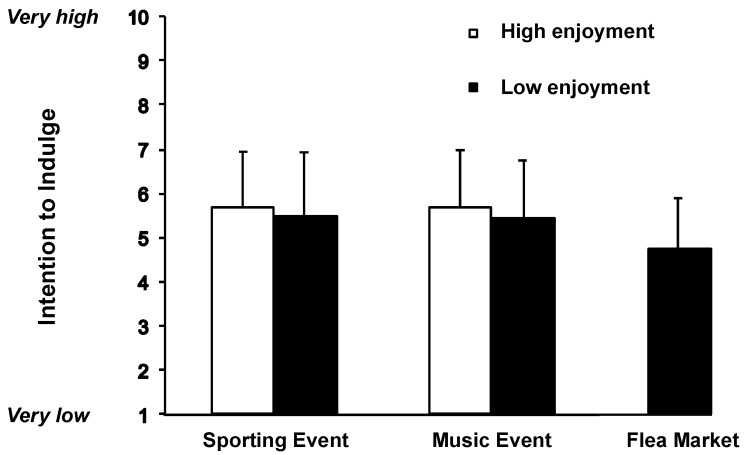
Intention to indulge in unhealthy foods versus healthy foods depending on the five experimental conditions: visit to a sporting event (low and high enjoyment), visit to a music event (low and high enjoyment), and visit to a flea market (low enjoyment only).

Figure 1 displays the participants’ intention to indulge in unhealthy foods versus healthy foods depending on the experimental conditions.

A linear regression analysis was performed to assess the influence of enjoyment (coded 1 for high and 0 for low) as well as the event (dummy 1, coded 1 for sporting event and 0 for other events; dummy 2, coded 1 for music event and 0 for other events) on the intention to indulge in unhealthy foods versus healthy foods. The variables explained 6% of the variance in the participants’ intention. While the influence of enjoyment was not significant (b [beta coefficient] = 0.23, SE [standard error] = 0.19, *p* = 0.22), both the visit to the sporting event (b = 0.67, SE = 0.26, *p* < 0.01) and the visit to the music event (b = 0.65, SE = 0.25, *p* < 0.01) increased the intention to indulge in unhealthy foods versus healthy foods. The contrast between sporting event and music event visits was not significant (b = 0.02, SE = 0.19, *p* = 0.93).

To conclude, it can be stated that, controlling for the influence of primed enjoyment of the visit, the attendance at a sporting event (versus a flea market) increased the intention to choose unhealthy foods versus healthy foods. The effect was similar for the attendance at a music event (versus a flea market), while the sporting event context and the music event context did not differ in their effects. We discuss the general implications below.

## 4. Discussion

The purpose of the study was to explore food- and drinks-related childhood memories about the attendance at sporting events and to find out whether sporting event attendance influences people’s preference for unhealthy foods versus healthy foods. The study contributes to the existing research in three ways.

First, the study revealed that eating and drinking contributed to an enjoyable stadium visit when adults reflected on their childhood experiences of spectator sports. Mostly unhealthy foods were recalled. While the provision of unhealthy foods at sporting events has been criticized [4,5,8], none of the previous studies has shown that childhood experiences with unhealthy foods are recalled even decades later nor how they shaped children’s food preferences. We note that any type of food can be part of a healthy, nutritious diet. However, when certain types of foods are consumed too often as well as in high amounts and when the consumption of high volumes of low-nutritious and high-caloric foods turns into a habit when eating at home or in other out-of-home contexts, the risk of children becoming overweight and obese may increase. The lived experience at sporting events may contribute to this, similarly to sponsorship-linked marketing activities of unhealthy food and drinks with role-model athletes as endorsers [7,9].

Second, the study revealed that both the sporting event context and the music event context are leisure-time activities that, controlling for enjoyment effects, increased the likelihood that individuals prefer unhealthy foods over healthy foods compared to a control group (here: people who imagined a visit to a flea market). The model explained 6% of the variance in the dependent variable. It is plausible that other factors than the venue influenced the preference for unhealthy versus healthy foods, such as individual taste preferences, social norms, and people’s general attitudes and values. Because of the random assignment of the participants to the various experimental conditions, however, the results should be unaffected by these individual differences. From a theoretical perspective, it is interesting that the two leisure-time activities—sporting event visit and music event visit—primed the intended food preferences of individuals. The potential reasons for this include that implicitly learned associations contribute to people preferring certain foods over others (such as eating hotdogs when watching a baseball game and eating popcorn when watching a movie in a cinema; [2]). Also, the anticipated convenience of certain foods may matter to food consumption intentions, depending on whether individuals assumed that seating was available or not, how much time was available, and how easy it was to dispose of left overs. Lastly, anticipated differences in social factors between contexts, such as crowding, which might be higher for music and sporting events than for a flea market, may have influenced the results.

Third, the finding that high (versus low) enjoyment of the event experience did not increase the preference for unhealthy and the avoidance of healthy food is noteworthy. On the one hand, one may have expected that individuals switch goals: when one goal (enjoyment) is achieved in the context of following the favorite sports team or music band, they may be directed to pursuing opposite goals (health goals in the context of eating) [25]. This was not the case in the present study. On the other hand, one may have expected that individuals want to nourish their consumption episode further: when they enjoy following their favorite sports team or music band, they want to enhance the enjoyment experience and continue to enjoy to have a ‘perfect evening’ (highlighting in the context of consumption episodes [26]). Although, in the present study, the means pointed in this direction (Figure 1), there were no significant effects in the analyses. Thus, the results did not support any of the two assumptions.

This study has important implications for public health policy and practice. There is a need for healthier food availability and food policy at sport settings in general and at sporting venues in particular. In the present study, all of the childhood memories about the association between food and sporting event attendance related to unhealthy foods (Study 1). Furthermore, the sporting context increased the preference for unhealthy over healthy foods (Study 2). To fight these links that promote unhealthy eating behaviors, public policy makers have several possibilities: (1) incentivize stakeholders to offer relatively more healthy food and drinks (such as providing funding to them if they offer sustainable, healthy options at sporting events); (2) make unhealthy (healthy) food and drinks less (more) accessible to sporting event visitors (e.g., via an increase in prices for unhealthy foods and drinks due to higher taxes); (3) make unhealthy (healthy) food and drinks less (more) attractive to sporting event visitors (e.g., via menu labeling or sponsorship of healthy food); (4) make unhealthy (healthy) food and drinks less (more) available by forced policy changes (this can range from forcing stakeholders to implement corporate social responsibility policies in relation to food and drinks to forbidding certain foods and drinks (e.g., high-sugar and high-fat options) per se in certain contexts, similarly to banning alcohol and smoking in sport stadiums). These measures may be most effective when both parents and children commit to the goal of healthy eating and drinking in sport settings [27] and when public policy-makers and practitioners include other stakeholders as well [28]. Consensus-based approaches are needed, because managers are afraid of loosing profits when strict policies are introduced [29].

The present study is not free of limitations. Study 1’s memorization task related to food and drinks that were available at the time that the informants recalled. Therefore, if healthy food and drink items were not offered to them, it is no surprise that they were not mentioned. Future studies should replicate the findings for sport fans that attended stadiums that offered about the same number of healthy and unhealthy food and drink options (so that there are equal chances to enter the recalled set of items), for example. Study 2’s experimental design controlled for enjoyment, but not for other contextual variables that may be relevant, such as convenience to eat food in relation to time and space. Also, the sample was skewed towards well-educated individuals, younger age, and females (versus males). Future studies may consider samples that are representative for stadium visitors. Future research may also look at the associations between (un)healthy food consumption and sporting event visits over time, as well as at choices depending on situational factors (such as an increase in the accessibility, attractiveness, and availability of healthy options; in particular: the introduction of menu-labeling schemes or when changes in family rituals in relation to food and drink consumption occur). While this study focused on memories in relation to healthy versus unhealthy food, future studies may focus on alcohol and tobacco consumption, factors that have also been identified as health threats to children and adults in sports settings [27,28,30].

## 5. Conclusions

To conclude, we can state that children should be exposed to healthy, but not unhealthy, food and drink environments when they attend sporting events as spectators, because these contextual factors and the memories related to the visits influence behavioral patterns later in life. The present study highlights the need for further action and further research in this important area.

## Figures and Tables

**Table 1:** Categories of childhood memories about the attendance of sporting events.

(Sub-)Category	Example Statement
Individual experiences	
Food and drink consumption	“I didn’t really like hotdogs that much, but for some reason they smelled so much better there.” (informant 11) “I was very happy and enjoying myself as I got a new hat, eating junk food, and learning about baseball. It seemed like a dream because everything was perfect.” (informant 20) “I remember vividly the taste of the hotdogs and excitedly watching the game in front of us. It is a highlight of my childhood.” (informant 26)
Emotions	“From the time the kick-off started until the final whistle, I did not sit down once. We thoroughly enjoyed the game.” (informant 4) “I enjoyed myself very much. It is a great day I loved to remember.” (informant 25)
Clothing	“We all wore green t-shirts.” (informant 2)
Merchandise	“I can almost see it now, sitting with my baseball cards from the gift shop (…) watching the game.” (informant 24)
Inspiration for life	“It was amazing to watch and experience as it taught me never give up even when things seem the bleakest.” (informant 29)
Nostalgia	“I still reflect back to the first time I stepped onto that hallowed ground, many, many years ago.” (informant 14)
External factors	
Game/show performance	“I was all excited to see my favorite player, Aurelio Rodriguez (third baseman).” (informant 18)
Built environment	“I was astonished by (…) the bowl-shaped facade.” (informant 14)
Weather	“It was so hot and muggy that day with no breeze or shade at all.” (informant 11)
Social factors	
Belongingness to family and friends	“It was a special day for me, and I’ll always remember seeing my first baseball game with my father and grandfather.” (informant 20)
Spectator-generated atmosphere	“The thrill of the audience was the best part.” (informant 25)

## Appendix A. Experimental Manipulations in Study 2

### Appendix A.1. Visit to a Sporting Event (High Enjoyment)

Please imagine that you attend a game of your favorite sports team. You have been looking forward to attending the game for months, and the media considers the game as the game of the year. Please read the brief description of how you feel on the day of the game. Please imagine how it would feel like to be there and to experience the game with all senses.

Before the game, you have met some of your family and friends and you drove to the stadium with them. You are in the stadium now and you cannot wait until the game begins. Your favorite team will play the biggest rival, and the game will have great influence on the league table. You can hear the fans already: they cheer for the team and there are amazing vibes in the stadium.

When you take your seat, the stadium, the fans, and the colorful flags that are all over the stadium overwhelm you. Your favorite players are on the pitch, and there is a tension in the air, anticipating that something special could occur today. You feel great, and everyone is ready to have a great evening, full of excitement and enjoyment. Nothing compares to this, and you have your ticket and are right there.

Before the game starts, you still have some time. Therefore, you want to get some food and drinks. The concession stands offer various food options. Please look at the following options and indicate the degree to which you would want to eat the respective food.

### Appendix A.2. Visit to a Sporting Event (Low Enjoyment)

Please imagine that you attend a game of your favorite sports team. You have been looking forward to attending the game, and the media broadcast the game. Please read the brief description of how you feel on the day of the game. Please imagine how it would feel like to be there and to experience the game with all senses.

Before the game, you have met some of your family and friends and you drove to the stadium with them. You are in the stadium now and you wait until the game begins. Your favorite team will play another team that is desperate to win the game. You cannot hear a lot of fans yet, because there are not many spectators inside the stadium. You hope that the stadium atmosphere will improve later.

When you take your seat, you try to get an overview of the stadium and look around in the visitor stands and on the pitch. You don’t see any of the players yet. You have to kill some time and think of what you could have done otherwise rather than attending this game. You still have to wait for the teams to enter the stadium and the game to begin.

Before the game starts, you still have some time. Therefore, you want to get some food and drinks. The concession stands offer various food options. Please look at the following options and indicate the degree to which you would want to eat the respective food.

### Appendix A.3. Visit to a Music Event (High Enjoyment)

Please imagine that you attend a concert of your favorite music band. You have been looking forward to attending the concert for months, and the media considers the concert as the concert of the year. Please read the brief description of how you feel on the day of the concert. Please imagine how it would feel like to be there and to experience the concert with all senses.

Before the concert, you have met some of your family and friends and you drove to the stadium with them. You are in the stadium now and you cannot wait until the concert begins. Your favorite band will host one of their greatest concerts, and the concert will feature songs from the new album. You can hear the fans already: they sing famous songs, and there are amazing vibes in the stadium.

When you take your seat, the stadium, the fans, and the colorful flags that are all over the stadium overwhelm you. Your favorite musicians are on the stage, and there is a tension in the air, anticipating that something special could occur today. You feel great, and everyone is ready to have a great evening, full of excitement and enjoyment. Nothing compares to this, and you have your ticket and are right there.

Before the concert starts, you still have some time. Therefore, you want to get some food and drinks. The concession stands offer various food options. Please look at the following options and indicate the degree to which you would want to eat the respective food.

### Appendix A.4. Visit to a Music Event (Low Enjoyment)

Please imagine that you attend a concert of your favorite music band. You have been looking forward to attending the concert, and the media broadcast the concert. Please read the brief description of how you feel on the day of the concert. Please imagine how it would feel like to be there and to experience the concert with all senses.

Before the concert, you have met some of your family and friends and you drove to the stadium with them. You are in the stadium now and you wait until the concert begins. Your favorite band will host a concert, and the concert will feature well-known and often-played songs. You cannot hear a lot of fans yet, because there are not many spectators inside the stadium. You hope that the stadium atmosphere will improve later.

When you take your seat, you try to get an overview of the stadium and look around in the visitor stands and on the stage. You don’t see any of the band members yet. You have to kill some time and think of what you could have done otherwise rather than attending this concert. You still have to wait for the band to enter the stadium and the concert to begin.

Before the concert starts, you still have some time. Therefore, you want to get some food and drinks. The concession stands offer various food options. Please look at the following options and indicate the degree to which you would want to eat the respective food.

### Appendix A.5. Visit to a Flea Market (Low Enjoyment)

Please imagine that you attend your favorite flea market. You have been looking forward to shop some items at the flea market. Many people visit this local flea market. Please read the brief description of how you feel on the day of the flea market. Please imagine how it would feel like to be there and to experience the flea market with all senses.

Before going to the flea market, you have met some of your family and friends and you drove to the place where it is held with them. You are at the market now and you wait until you find a good bargain or an item that you like. The market features some well-known stands, and people regularly purchase from these stands. There are not a lot of visitors at the flea market yet, and you hope that the atmosphere will improve later.

When you stroll around, you try to get an overview of the market and look around among people and what the stands have to offer. You don’t see anything exciting yet. You have to kill some time and think of what you could have done otherwise rather than attending this flea market. You still want to find one or two items that you can bring home with you.

Before this and before you go home, you still have some time. Therefore, you want to get some food and drinks. The concession stands offer various food options. Please look at the following options and indicate the degree to which you would want to eat the respective food.

## Appendix B. Foods Used in Study 2

The following foods were used (R indicates reverse coding): Asia soup (R), burger, chicken wings, chocolate muffin, french fries, fried onion rings, fruit salad (R), hotdog, meat skewers, pizza, salad (R), vegetable quiche (R), vegetable soup (R), vegetable wrap (R), and yogurt (R). The foods were displayed in a typical serving size and in a way so that they could be readily eaten by individuals while sitting or standing, on a disposable plate or in a cup/bowl.
